# Is the adult EBMT risk score valid and relevant for hematopoietic stem cell transplantation in children acute leukemia?

**DOI:** 10.1038/s41409-026-02952-8

**Published:** 2026-07-20

**Authors:** F. Baleydier, J. E. Galimard, F. Bernard, F. Gonzales, A. Dalissier, A. Al-Seraihy, J.-H. Dalle, C. R. S. Uppugunduri, L. Zubarovskaya, M. Zecca, F. Locatelli, A. Kulagin, F. Fagioli, S. Robinson, A. Balduzzi, A. Biffi, Y. Chalandon, P. Bader, S. Corbacioglu, Krzysztof Kalwak, M. Ansari

**Affiliations:** 1 Paediatric Onco-Haematology Unit, Geneva University Hospital, Geneva, Switzerland; 2 Cansearch Research platform for pediatric oncology and hematology, Faculty of Medicine, Department of Pediatrics, Gynecology and Obstetrics, University of Geneva, Geneva, Switzerland; 3 EBMT Paris Study Unit, Paris, France; 4 King Faisal Specialist Hospital & Research Centre, Jeddah, Saudi Arabia; 5 Department of Paediatric Haematology and immunology, Robert Debré Hospital, GHU AP-HP Nord, Université Paris Cité, Paris, France; 6 Department of Medical Oncology, Jawaharlal Institute of Postgraduate Medical Education and Research, Puducherry, India; 7 RM Gorbacheva Research Institute, Pavlov University, St. Petersburg, Russia; 8 Pediatric Hematology-Oncology, Fondazione IRCCS Policlinico San Matteo, Pavia, Italy; 9 IRRCS Ospedale Pediatrico Bambino Gesù, Sapienza University of Rome, Rome, Italy; 10 Catholic University of the Sacred Heart, Rome, Italy; 11 Onco-Ematologia Pediatrica Centro Trapianti Cellule Staminali, Torino, Italy; 12 Department of Paediatric Oncology/BMT, Bristol Royal Hospital for Children, Bristol, UK; 13 Pediatric Hematopoietic Stem Cell Transplant Unit, Fondazione IRCCS San Gerardo dei Tintori, Monza, Italy; 14 Department of Medicine and Surgery, University of Milano-Bicocca, Milano, Italy; 15 Division of Pediatric Hematology, Oncology and Stem Cell Transplantation, University of Padova, Padova, Italy; 16 Haematology division and Bone Marrow Transplantation Unit, Geneva University Hospital, Geneva, Switzerland; 17 Division for Stem Cell Transplantation and Immunology, University Hospital Frankfurt, Goethe University, Frankfurt am Main, Germany; 18 Department of Paediatric Haematology, Oncology and Stem Cell Transplantation, University Hospital of Regensburg, Regensburg, Germany; 19 Clinical Department of Pediatric BMT, Hematology and Oncology, Wroclaw Medical University, Wroclaw, Poland

**Keywords:** Haematological cancer, Medical research

## Abstract

The EBMT risk score, incorporating five factors to estimate the risks associated with haematopoietic stem cell transplantation (HSCT) in adult patients, has not been validated in children. This study aims to assess this score in 9447 children undergoing allogeneic HSCT for acute lymphoblastic or myeloid leukemia (ALL/AML) between 2010 and 2021. Risk factors were coded according to the original EBMT score. In both ALL (*n* = 5774) and AML (*n* = 3673), only disease status and the time from diagnosis to HSCT significantly impacted all the outcomes in the multivariable analysis. The combination of female donors and male recipients did not affect overall survival (OS), relapse incidence (RI), non-relapse mortality (NRM), and leukemia-free survival (LFS). Additionally, the prognostic impact of donor type was not evident; when viewed as two modalities, the impact of MSD versus non-MSD on different outcomes in ALL was inconsistent with those observed in AML. Consequently, in both ALL and AML, scores ranging from 3 to 5 significantly affected all outcomes, whereas a score of 2 did not significantly impact NRM or RI in AML. Therefore, this analysis demonstrates that the adult EBMT risk score is not entirely reliable in children with acute leukemia, highlighting the need for a new pediatric-specific prognostic score.

## Introduction

In 1998, the European Group for Blood and Marrow Transplantation (EBMT) developed a pre-transplant risk score for patients with chronic myeloid leukemia eligible for haematopoietic stem cell transplantation (HSCT) [1]. The goal was to provide a tool able to accurately predict the risk of transplantation-related morbidity and mortality. This score remains one of the easiest among the proposed scoring systems [2]. It is based on five pre-transplant parameters, including donor type, disease status at transplantation, recipient age, donor-recipient sex combination, and the time from diagnosis to transplant. These five parameters had an independent and significant impact on leukemia-free survival (LFS), overall survival (OS), and transplantation-related mortality (TRM).

After further validation in larger cohorts of patients, this score was extended to many other acquired malignant and non-malignant hematological disorders and is now used routinely in adult centers [3, 4]. In pediatrics, albeit some centers use the adult EBMT risk score, the challenges of HSCT are different from those in adults [5]. In inherited disorders and other non-malignant conditions, which represent a significant proportion of transplant indications in pediatrics, TRM must typically be carefully assessed in regard to the non-fatal outcome of some of these diseases [5]. Additionally, for leukemias, age categories correlate more closely with leukemia-related risk factors [6, 7] than with age-related comorbidities, which may, however, be connected more to preexisting inherited conditions. Thirdly, pediatric transplanters must primarily prioritize curative options regarding the life expectancy of their patients; therefore, they are inclined to consider more aggressive procedures such as HSCT, even in challenging cases.

Therefore, although a pretransplant risk score could also benefit pediatric patients, using the adult EBMT risk score brings its reliability in pediatric HSCT into question. Thus, to address this issue, we preliminarily undertook to evaluate the adult EBMT risk score on a cohort of pediatric transplant patients registered in the EBMT database who were affected by either acute lymphoblastic leukaemia (ALL) or acute myeloid leukaemia (AML), the two primary indications for HSCT in pediatrics [5, 8].

## Material and methods

This study is a retrospective registry-based analysis conducted on behalf of the Pediatric Diseases Working Party (PDWP) of the EBMT. All patients presenting with acute leukemia and aged under 18 years at the time of transplantation who underwent first allogeneic HSCT between 2010 and 2021 were analyzed. Informed consent from the patients and/or their relatives was obtained before enrolment into the EBMT registry.

The aim was to evaluate the prognostic value of the EBMT risk score on the outcomes in the pediatric population. Quantitative variables were described using the median, first quartile, third quartile, minimum, and maximum values. Qualitative variables were presented as counts and percentages. Outcomes included OS, LFS, relapse incidence (RI), non-relapse mortality (NRM), acute graft-versus-host disease (GVHD), and GVHD-free Relapse-free survival (GRFS) [9]. LFS was calculated from HSCT until the date of first relapse, death from any cause, or the last follow-up. NRM was defined as death in the absence of relapse, while GRFS was calculated as the time elapsing between grade III-IV acute GVHD, extensive chronic GVHD, relapse, or death, whichever occurred first.

OS, LFS, and GRFS were estimated using the Kaplan- Meier method. Cumulative incidence functions were employed to estimate the incidence of relapse, NRM, acute GVHD, and chronic GVHD. Competing risk for relapse was NRM, while for NRM, relapse was the competing risk. Relapse and death represented competing events for acute and chronic GVHD. Multivariable analyses were conducted using the Cox proportional hazard model. Except for age, since all patients were under 18 years and consequently assigned to a score of zero (same category as in the EBMT risk score), the variables used to calculate the EBMT risk score (disease status at transplant, time between diagnosis and transplant, donor type, and female donor to male recipients) were assessed as coded in the adult EBMT score (see Table 1) established by Gratwohl et al. for adult malignant and non-malignant hematological diseases, including ALL and AML [3, 4].

**Table 1:** Adult derived pediatric EBMT scoring.

Adult		Pediatric	
Parameter	EBMT risk score	Parameter	EBMT risk score
Age (years)		Age (years)	
<20	0	0-2	0
20-40	1	2-4	0
>40	2	4-10	0
		10-<18	0
Disease status		Disease status / time from diagnostic to HSCT	
Early	0	CR1	0
Intermediate	1	CR2*	1
Late	2	CR3^§^	2
Time interval from diagnostic to transplant		Time interval from diagnostic to transplant	
<12 months	0	<12 months	0
>12 months	1	>12 months	1
Donor type		Donor type	
HLA-identical sibling donor	0	HLA-identical sibling donor	0
Unrelated donor, other	1	Unrelated donor, other	1
Donor recipient sex combination		Donor recipient sex combination	
All other	0	All other	0
Female donor to male recipient	1	Female donor to male recipient	1

*equivalent to intermediate disease stage in the adult EBMT score.

^§^equivalent to late disease stage in the adult EBMT score.

All aforementioned variables were included in a multivariable Cox regression model. A second model incorporated only the EBMT risk score as a qualitative variable. To account for possible center effects, we introduced a random effect (also known as a frailty effect) for each center. Analyses were conducted separately for AML and ALL. The significance level was set at .05, and *P* values were two-sided. Statistical analyses were performed using R software version 3.2.3 (R Development Core Team, Vienna, Austria).

## Results

The 9447 pediatric patients, comprising 5774 with ALL and 3673 with AML, registered in the EBMT registry from 2010 to 2021, were extracted for this study. Descriptive statistics for the cohort are summarized in supplementary Table 1. Forty-seven percent of patients were transplanted in the first complete remission (CR1), 39% in CR2, and 14% in CR3 or more advanced disease. All outcomes are detailed in supplementary Table 2. Briefly, the median follow-up was 3 years (95% CI 3–3.1) for the entire cohort, and 2.9 years (95% CI 2.8–3) and 3.2 years (95% CI 3.1–3.4) for ALL and AML patients, respectively. For the entire cohort, three-year LFS and OS were 57.3%, (95% CI 56.2–58.4), and 65.5%, (95% CI 64.4–66.6), respectively; three-year RI and NRM were 30%, (95% CI 29–31.1) and 12.7%, (95% CI 12–13.4), respectively.

Overall, the three-year LFS and OS in ALL were 56.3%, (95% CI 54.8–57.7) and 65.5%, (95% CI 64.1–66.9), respectively (see supplementary Table 2). Concerning disease status at transplantation, overall, 37%, 48% and 15% were transplanted in CR1, CR2 and CR3 or more advanced disease, respectively (see supplementary Table 2). The status of disease at transplantation combined with the time from diagnosis to transplant significantly impacted OS, LFS, RI and GRFS in the multivariable analysis (*p* < 0.001; see supplementary Table 3). Focusing on CR2 patient group, unlike for CR3 and active disease patient group, those transplanted more than 12 months after the diagnosis showed a trend toward better outcomes than those transplanted during the first 12 months post-diagnosis (see supplementary Table 3). With its significant impact on acute and chronic GVHD, donor type significantly influenced OS, LFS, NRM and GRFS but not RI (see supplementary Table 3). In pediatric ALL patients, the female donor to male recipient combination did not influence OS, LFS, RI, NRM and GRFS but significantly impacted both grade III-IV acute and chronic GVHD (see supplementary Table 3).

In AML, three-year LFS and OS were 58.8% (95% CI 57.1–60.6) and 65.5% (95% CI 63.7–67.2), respectively (see supplementary Table 2). The proportion of patients transplanted in CR1, CR2, and CR3 or more advanced disease was 62%, 26%, and 12%, respectively, a proportion quite different from that observed in ALL (see supplementary Table 1). Compared to CR1, the significant influence of disease status at HSCT, combined with the time from diagnosis to transplant, was still observed on OS, LFS, RI, and GRFS for all patients with the exception of those presenting with CR2 status who underwent HSCT more than 12 months after the primary diagnosis and who had a prognosis similar to that of children transplanted in CR1. The significant prognostic impact of donor type remained present for NRM and acute GVHD, but not for chronic GVHD or malignancy-related outcomes. As we observed in ALL, unlike in adult patients, the female donor to male recipient combination did not impact any outcome except for significantly affecting the incidence of chronic and chronic-extensive GVHD.

**Fig. 1:**
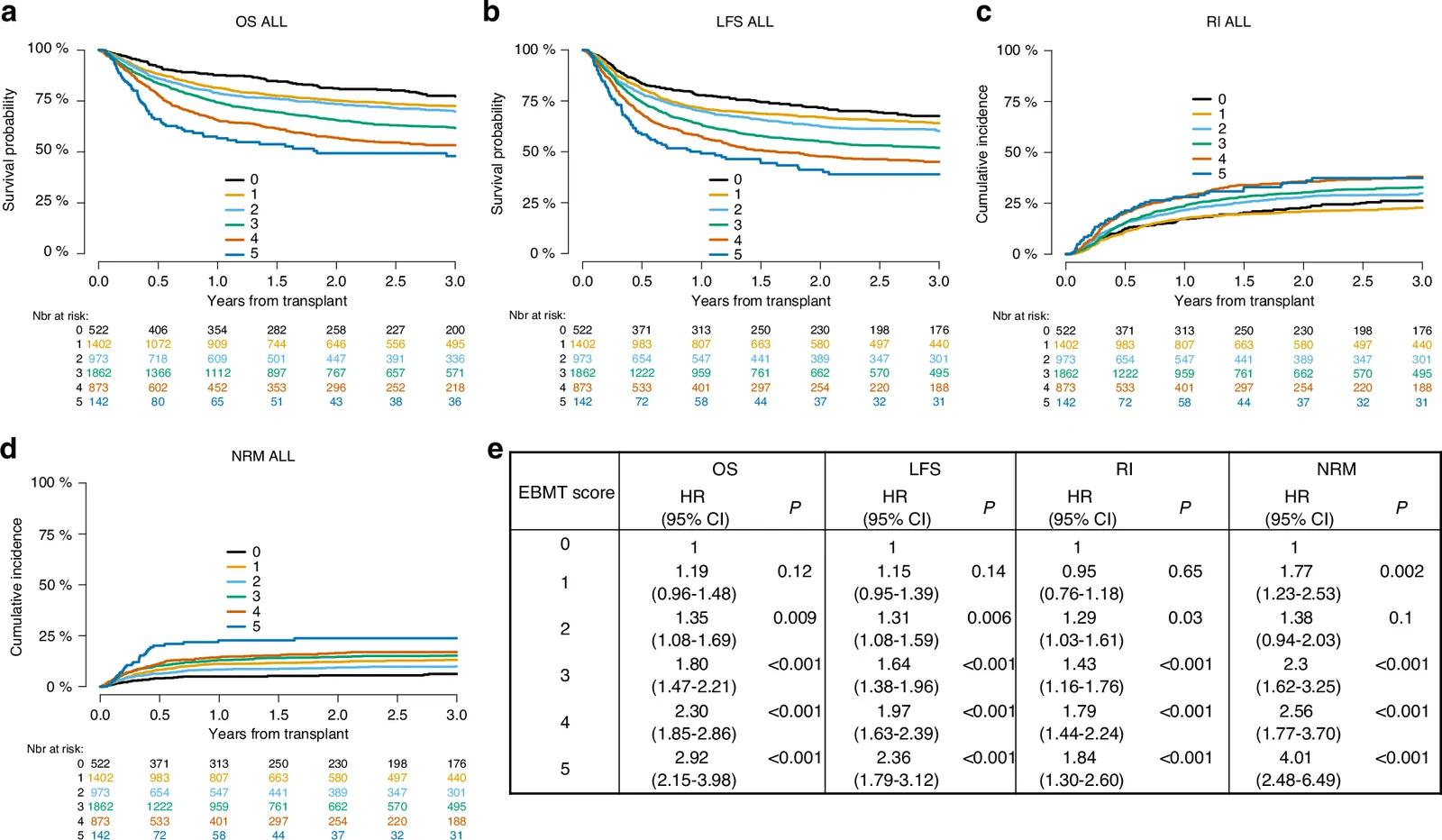
Outcomes of transplanted ALL patients according to the EBMT risk score. **a, b, c, d** correspond to overall survival (OS), leukemia-free survival (LFS), relapse incidence (RI), and non-relapse mortality (NRM), respectively. **e** summarizes the hazard ratio for each outcome according to EBMT score values and their corresponding *P*-value. EBMT score equal to or higher than two is significantly associated with impaired survival outcomes. Moreover, patients with a score equal to or higher than two and with a score equal to 1 or ranged from 3 to 5, have a significantly higher risk of RI and NRM, respectively.

**Fig. 2:**
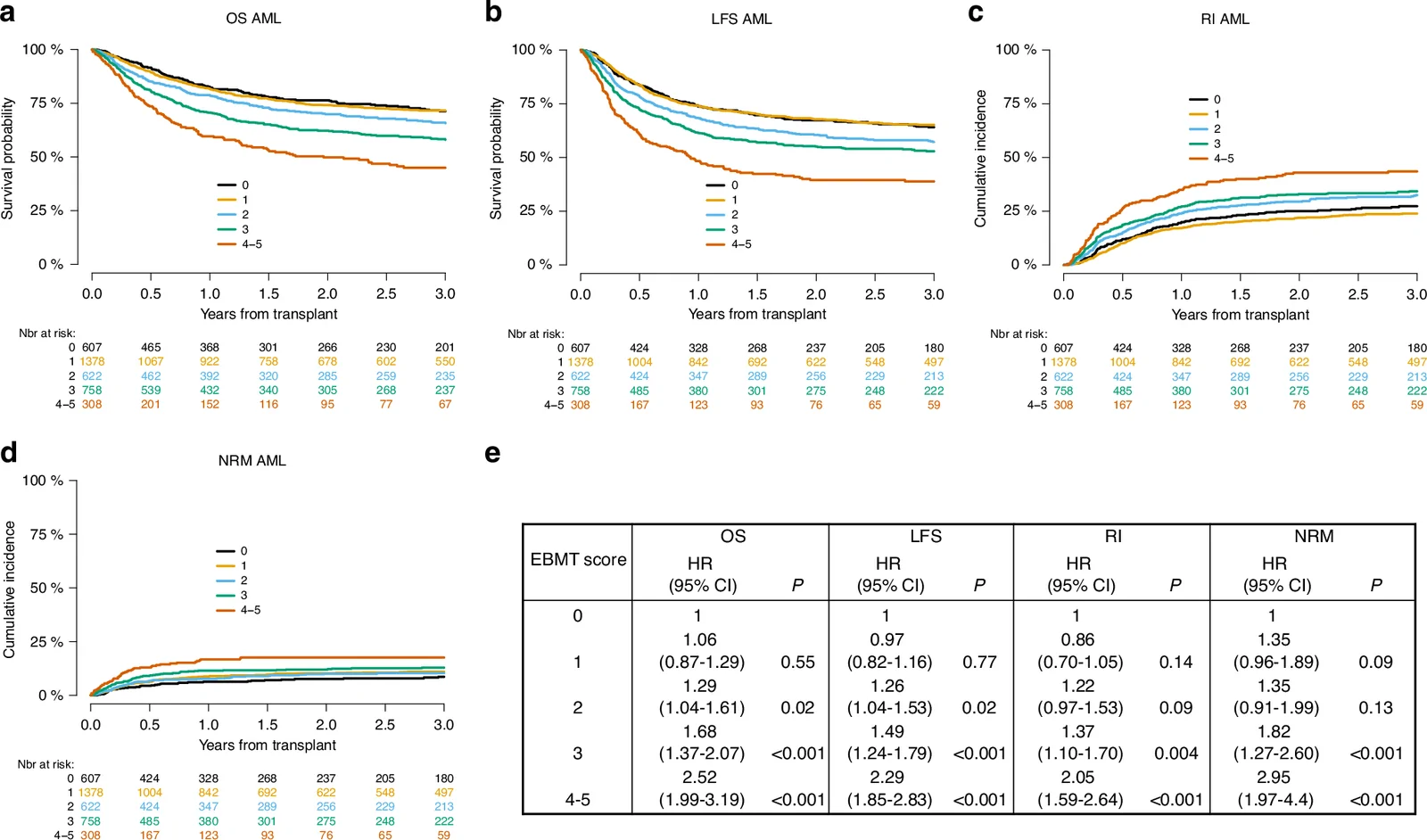
Outcomes for transplanted AML patients according to the EBMT score. **a, b, c, d** represent overall survival (OS), leukemia-free survival (LFS), relapse incidence (RI), and non-relapse mortality. **e** summarizes the hazard ratio for each outcome according to EBMT score values and their corresponding *P*-value. The same results are observed as for ALL patients. Survival outcomes are significantly worsened for scores equal to or higher than two, while RI and NRM are significantly higher in patients with score higher than three.

The adult EBMT scoring system applied to our pediatric cohort ranged from 0 to 5. In ALL, a score from 0 to 5 was associated with a significant decrease (*p* < 0.001) in three-year LFS from 68%, (95% CI 62.7–72) to 44%, (95% CI 40.7–47.6), and a significant decrease (*p* < 0.001) in three- year OS from 77%, (95% CI 72.6–81.2) to 53%, (95% CI 49–56.1) (see Fig. 1a, b, e). The only outcome significantly affected by a score of 1 was NRM (see Fig. 1d, e) due to its significant impact on the risk of acute GVHD (see Table 2). A score of 2 had a significant impact on OS, LFS, RI, and GRFS but not on NRM. Scores between 3 and 5 negatively affected all outcomes (see Table 2 and Fig. 1a–d, e). In AML, a score of 1 did not affect any outcomes and was only linked to a significant risk of grade III-IV acute GVHD (see Table 3). A score of 2 significantly impaired three-year OS and LFS (see Fig. 2c, d, e). Scores higher than 2 significantly worsened all outcome parameters (see Table 3). For scores of 3 and 4-5 combined, three-year LFS dropped from 64%, (95% CI 59.4–68.3), to 39%, (95% CI 32.9–44.8), respectively (Fig. 2c, d) while three-year RI increased from 27%, (95% CI 23.3–31.5), to 44%, (95% CI 37.5–49.5), respectively. During the decade covered by our study, significant improvements occurred in leukemia treatment, conditioning regimens, and allogeneic-HSCT supportive care. Therefore, we tested the outcome according to the EBMT score after splitting the cohort into the two periods of time, 2010–2014 and 2015–2021. Across these two periods, LFS slightly improved for both ALL and AML (Supplementary Fig. 1) during the late 2015–2021 period compared with the early 2010–2014 period. For ALL, the EBMT score seems less powerful in discriminating LFS during the late 2015–2021 era (Supplementary Fig. 1a, b). For AML, scores ranging from 2 to 4–5 appear more powerful in discriminating LFS during the late 2015–2021 period (Supplementary Fig. 1c, d).

**Table 2:** Analysis of outcome parameters of acute lymphoblastic leukemia according to EBMT score.

EBMT score	OS HR (95% CI) p	LFS HR (95% CI) p	RI HR (95% CI) p	NRM HR (95% CI) p	Acute III-IV GVHD HR (95% CI) p	Chronic GVHD HR (95% CI) p	GRFS HR (95% CI) p
0	1	1	1	1	1	1	1
1	1.19 (0.96–1.48)	1.15 (0.95–1.39)	0.95 (0.76–1.18)	1.77 (1.23–2.53)	1.67 (1.16–2.39)	1.23 (0.91–1.65)	1.17 (0.99–1.38)
	0.12	0.14	0.65	0.002	0.006	0.17	0.07
2	1.35 (1.08–1.69)	1.31 (1.08–1.59)	1.29 (1.03–1.61)	1.38 (0.94–2.03)	1.57 (1.07–2.30)	1.29 (0.95–1.76)	1.35 (1.14–1.60)
	0.009	0.006	0.03	0.1	0.02	0.11	<0.001
3	1.80 (1.47–2.21)	1.64 (1.38–1.96)	1.43 (1.16–1.76)	2.3 (1.62–3.25)	1.64 (1.15–2.33)	1.44 (1.08–1.92)	1.59 (1.36–1.86)
	<0.001	<0.001	<0.001	<0.001	0.007	0.01	<0.001
4	2.30 (1.85–2.86)	1.97 (1.63–2.39)	1.79 (1.44–2.24)	2.56 (1.77–3.70)	1.87 (1.28–2.73)	1.40 (1.02–1.93)	1.77 (1.49–2.09)
	<0.001	<0.001	<0.001	<0.001	<0.001	0.04	<0.001
5	2.92 (2.15–3.98)	2.36 (1.79–3.12)	1.84 (1.30–2.60)	4.01 (2.48–6.49)	1.90 (1.06–3.39)	1.73 (1.01–2.96)	2.10 (1.62–2.72)
	<0.001	<0.001	<0.001	<0.001	0.03	0.04	<0.001

*LFS* leukemia-free survival, *RI* relapse incidence, *NRM* non-relapse mortality, *GRFS* GVHD-relapse-free survival, *OS* overall survival, *GVHD* graft-versus-host disease.

**Table 3:** Analysis of outcome parameters of acute myeloid leukemia according to EBMT score.

EBMT score	OS	LFS	RI	NRM	Acute III-IV GVHD	Chronic GVHD	GRFS
	HR (95% CI) p	HR (95% CI) p	HR (95% CI) p	HR (95% CI) p	HR (95% CI) p	HR (95% CI) p	HR (95% CI) p
0	1	1	1	1	1	1	1
1	1.06 (0.87–1.29)	0.97 (0.82–1.16)	0.86 (0.70–1.05)	1.35 (0.96–1.89)	1.51 (1.09–2.10)	1.31 (0.99–1.73)	1.13 (0.96-1.33)
	0.55	0.77	0.14	0.09	0.01	0.06	0.13
2	1.29 (1.04–1.61)	1.26 (1.04–1.53)	1.22 (0.97–1.53)	1.35 (0.91–1.99)	1.19 (0.81–1.76)	1.31 (0.95–1.82)	1.25 (1.05-1.50)
	0.02	0.02	0.09	0.13	0.38	0.1	0.01
3	1.68 (1.37–2.07)	1.49 (1.24–1.79)	1.37 (1.10–1.70)	1.82 (1.27–2.60)	1.71 (1.20–2.44)	1.66 (1.22–2.25)	1.52 (1.28-1.80)
	<0.001	<0.001	0.004	<0.001	0.003	<0.001	<0.001
4–5	2.52 (1.99–3.19)	2.29 (1.85–2.83)	2.05 (1.59–2.64)	2.95 (1.97–4.4)	1.64 (1.06–2.56)	1.61 (1.07–2.4)	2.11 (1.73–2.57)
	<0.001	<0.001	<0.001	<0.001	0.03	0.02	<0.001

## Discussion

This study aimed to evaluate the impact of the adult EBMT risk score in a pediatric population presenting with acute leukemia who undergo allogeneic-HSCT. We demonstrated that the adult EBMT risk score is also predictive of the outcomes in pediatric HSCT, but its relevance in this population remains uncertain. Although some variations exist according to conditions, type of donor, and conditioning regimen, the cumulative incidence of NRM for pediatric and adult HSCT has substantially decreased over the last decades [8]. However, the benefit/risk ratio of HSCT differs significantly between pediatric and adult patients [8]. For example, the age of the patient, one of the five items of the adult EBMT score, is of major importance in adult patients, typically for elderly patients in whom the benefit/risk ratio of HSCT should be carefully balanced with age-related co-morbidities [8]. On the contrary, in children, age-related co-morbidities carry less weight, while certain underlying inherited conditions or the disease status at HSCT may have greater implications. Indeed, although worse NRM has been reported in patients older than 10 years [10] or, alternatively, more than 16 years of age [5], the meaning and relevance of age in pediatric BMT scoring is more reflective of unfavorable prognostic factors of the leukemia [6, 11], rather than underlying co-morbidities that subsequently limit therapeutic achievement [12, 13]. Therefore, we believe that the age parameter should not be considered for either a pediatric scoring system or for decisions regarding HSCT in pediatrics. This work further shows that, as expected, the disease status at transplantation remains the strongest parameter affecting the prognosis of HSCT for acute leukemias [14–16]. However, HSCT in pediatric acute leukemias only accounts for about 50% of pediatric HSCT cases [17], and is usually indicated only in the absence of alternative valid options. Further, depending on disease status and the disease itself, the timing from diagnosis to HSCT has varying impacts between children with ALL and AML, as well as between children and adults, in whom, unlike children who are in CR2, a longer time from diagnosis to transplant appears to significantly worsen the outcome [3, 4]. The problem of this parameter and the choice of the 12-month cutoff have been extensively discussed by Gratwohl et al. [3]. Briefly, dual meaning of shorter versus longer time from diagnosis to transplant in a setting of relapse may be summarized as follows: short time may reflect the aggressiveness of a disease relapsing early or in contrast a disease requiring relatively slight treatment to obtain a good CR2/CR3 before HSCT; long time may sign late relapse of a less aggressive disease or in contrast, a disease requiring more treatment, and therefore more pre-transplant toxicity, to obtain CR2/CR3. In the future, it is likely that the extensive use of immunotherapy as a bridge-to-transplant will decrease even more the weight of this parameter, at least in ALL. In children, we have shown that the type of donor has differing impacts between AML (no prognostic influence) and ALL (where the use of an unrelated donor was associated with worse OS and LFS), which contrasts with what is observed in adults, where donor type has a stronger impact in AML than in ALL [3]. Regarding the female donor to male recipient combination, in children, this parameter does not significantly affect any outcomes, while it does in adults, although this impact is lesser in adult ALL and AML compared to other diseases considered [3]. Thus, in its current configuration, the EBMT risk score used for adults does not seem particularly relevant in pediatrics. The Bone-Marrow Transplantation pediatric UK group from Great Ormond Street Hospital made the same observation when they attempted to validate the adult HCT-CI score in their pediatric cohort [18]. They subsequently tailored a specific pediatric predictive score for post-HSCT outcomes, the Pediatric Adapted Risk Index (PARI) [19]. In our score, beyond the issue of age, we show that only the status of the disease combined with the time from diagnosis to transplant robustly predicts the outcome. LFS improvement according to the EBMT score observed in both ALL and AML during the late 2015–2021 area support that point. Indeed, especially in ALL, it is likely that the successful use of immunotherapies in bridge-to-transplant during this period has produced better control of the disease before transplantation and subsequent improvement of disease status. This is consistent with many reports demonstrating that disease status at transplantation is widely recognized as the major prognostic factor significantly impacting LFS [16, 20–22]. Thus, M. Qayed et al. developed a disease risk index for allo-HSCT in pediatric AML and ALL mainly based on disease status before transplant [23]. Moreover, unlike in the PARI, pretransplant comorbidities are not taken into account in the adult EBMT score but seem to be determinants in predicting 2-year NRM in pediatrics, especially as pre-transplant infectious events along with lung and central nervous system diseases [19]. In current pediatric practice, those comorbidities are indeed widely considered before deciding on BMT. Therefore, pediatric hematologists are unlikely to make the decision to offer HSCT for high-risk leukemias solely based on the adult EBMT risk score. Particularly, with emerging bridge-to-transplant strategies that avoid chemotherapies, typically such as immunotherapies, they will probably be much more inclined to undertake HSCT after evaluating measurable residual disease at transplantation in combination with past and/or ongoing significant toxicities/comorbidities resulting from previous treatments and/or potential underlying inherited conditions, especially those predisposing to cancer. Moreover, this study aimed to gauge the accuracy of the adult EBMT score in predicting outcome after HSCT in children presenting with acute leukemia, whereas about 50% of HSCT in children are performed for diseases other than malignancies, such as inherited immunological or benign hematological disorders [17]. This raises concerns about a potentially life-threatening procedure in a non-malignant disease, which may benefit from a scoring system to help the decision-making process. Unfortunately, none of the parameters from the adult EBMT score that are not related to disease status at transplantation demonstrate a robust predictive effect on outcome and, therefore, could not be considered useful for scoring in diseases other than leukemia.

In addition to the limitations already mentioned above, such as the absence of age scoring for pediatric patients and analysis restricted to leukemia patients, one more limitation is the absence of an intention-to-treat concept since only effectively transplanted patients are registered in our database. Indeed, we can not exclude that some centers have abandoned HSCT for patients they judged to not fit this treatment enough.

In conclusion, having a risk score for pediatric HSCT that predicts outcomes similar to those of adults would be obviously useful. However, applying the adult risk score to pediatric patients is hampered by substantial limitations. The age factor should be removed from the parameters and re-evaluated in a different way. Moreover, the impact of certain parameters differs between adults and children, with the strongest predictive factor primarily being the disease status at transplantation. Consequently, a pediatric-specific EBMT scoring system that reflects the wider variety of diseases or disease-specific individualized scoring systems should be identified and validated to accurately predict the outcomes for pediatric patients.

## Supplementary information

Supplementary figure legend

Supplementary Table 1

Supplementary Table 2

Supplementary Table 3

Supplementary Figure 1

## Data Availability

All the data presented in this manuscript and used for analyses were extracted from the EBMT registry database (https://www.ebmt.org/registry/ebmt-registry). Any request regarding the dataset used for this work can be addressed to Jacques-Emmanuel Galimard at jacques-emmanuel.galimard@ebmt.org.

## References

[1] Gratwohl A, Hermans J, Goldman JM, Arcese W, Carreras E, Devergie A, et al. Risk assessment for patients with chronic myeloid leukaemia before allogeneic blood or marrow transplantation. Chronic Leukemia Working Party of the European Group for Blood and Marrow Transplantation. Lancet. 1998;352:1087–92. 10.1016/s0140-6736(98)03030-x 9798583

[2] Potdar R, Varadi G, Fein J, Labopin M, Nagler A, Shouval R. Prognostic scoring systems in allogeneic hematopoietic stem cell transplantation: where do we stand? Biol Blood Marrow Transpl. 2017;23:1839–46. 10.1016/j.bbmt.2017.07.028 28797781

[3] Gratwohl A. The EBMT risk score. Bone Marrow Transpl. 2012;47:749–56. 10.1038/bmt.2011.110 21643021

[4] Gratwohl A, Stern M, Brand R, Apperley J, Baldomero H, de Witte T, et al. Risk score for outcome after allogeneic hematopoietic stem cell transplantation: a retrospective analysis. Cancer. 2009;115:4715–26. 10.1002/cncr.24531 19642176

[5] Miano M, Labopin M, Hartmann O, Angelucci E, Cornish J, Gluckman E, et al. Haematopoietic stem cell transplantation trends in children over the last three decades: a survey by the paediatric diseases working party of the European Group for Blood and Marrow Transplantation. Bone Marrow Transpl. 2007;39:89–99. 10.1038/sj.bmt.1705550 17213848

[6] Takachi T, Watanabe T, Miyamura T, Moriya Saito A, Deguchi T, Hori T, et al. Hematopoietic stem cell transplantation for infants with high-risk KMT2A gene-rearranged acute lymphoblastic leukemia. Blood Adv. 2021;5:3891–9. 10.1182/bloodadvances.2020004157 PMC8679679 34500465

[7] van Weelderen RE, Klein K, Harrison CJ, Jiang Y, Abrahamsson J, Arad-Cohen N, et al. Measurable residual disease and fusion partner independently predict survival and relapse risk in childhood KMT2A-rearranged acute myeloid leukemia: a study by the International Berlin-Frankfurt-Münster Study Group. J Clin Oncol. 2023;41:2963–74. 10.1200/JCO.22.02120 PMC10414713 36996387

[8] Sureda A, Corbacioglu S, Greco R, Kroger N, Carreras E, editors. The EBMT handbook: hematopoietic cell transplantation and cellular therapies. 8th ed. Gewerbestrasse 11, 6330 Cham, Switzerland: Springer Nature; 2024. 39437029

[9] Mehta RS, Holtan SG, Wang T, Hemmer MT, Spellman SR, Arora M, et al. GRFS and CRFS in alternative donor hematopoietic cell transplantation for pediatric patients with acute leukemia. Blood Adv. 2019;3:1441–9. 10.1182/bloodadvances.2018030171 PMC6517657 31053571

[10] Woolfrey AE, Anasetti C, Storer B, Doney K, Milner LA, Sievers EL, et al. Factors associated with outcome after unrelated marrow transplantation for treatment of acute lymphoblastic leukemia in children. Blood. 2002;99:2002–8. 10.1182/blood.v99.6.2002 11877272

[11] Meyer C, Larghero P, Almeida Lopes B, Burmeister T, Groger D, Sutton R, et al. The KMT2A recombinome of acute leukemias in 2023. Leukemia. 2023;37:988–1005. 10.1038/s41375-023-01877-1 PMC10169636 37019990

[12] Bader P, Potschger U, Dalle JH, Moser LM, Balduzzi A, Ansari M, et al. Low rate of nonrelapse mortality in under-4-year-olds with ALL given chemotherapeutic conditioning for HSCT: a phase 3 FORUM study. Blood Adv. 2024;8:416–28. 10.1182/bloodadvances.2023010591 PMC10827403 37738088

[13] Vrooman LM, Millard HR, Brazauskas R, Majhail NS, Battiwalla M, Flowers ME, et al. Survival and late effects after allogeneic hematopoietic cell transplantation for hematologic malignancy at less than three years of age. Biol Blood Marrow Transpl. 2017;23:1327–34. 10.1016/j.bbmt.2017.04.017 PMC5666571 28461213

[14] Balduzzi A, Glogova E, Peters C, Sedlacek P, Dalle JH, Locatelli F, et al. Impact of minimal residual disease on the outcome of hematopoietic stem cell transplantation for childhood acute lymphoblastic leukemia within the FORUM Trial. Haematologica. 2025;111:122–34. 10.3324/haematol.2025.287456 PMC12775767 40820816

[15] Balduzzi A, Di Maio L, Silvestri D, Songia S, Bonanomi S, Rovelli A, et al. Minimal residual disease before and after transplantation for childhood acute lymphoblastic leukaemia: is there any room for intervention? Br J Haematol. 2014;164:396–408. 10.1111/bjh.12639 24422724

[16] Walter RB, Buckley SA, Pagel JM, Wood BL, Storer BE, Sandmaier BM, et al. Significance of minimal residual disease before myeloablative allogeneic hematopoietic cell transplantation for AML in first and second complete remission. Blood. 2013;122:1813–21. 10.1182/blood-2013-06-506725 PMC3765060 23847197

[17] Snowden JA, Sanchez-Ortega I, Corbacioglu S, Basak GW, Chabannon C, de la Camara R, et al. Indications for haematopoietic cell transplantation for haematological diseases, solid tumours and immune disorders: current practice in Europe, 2022. Bone Marrow Transpl. 2022;57:1217–39. 10.1038/s41409-022-01691-w PMC9119216 35589997

[18] Wood W, Deal A, Whitley J, Sharf A, Serody J, Gabriel D, et al. Usefulness of the hematopoietic cell transplantation-specific comorbidity index (HCT-CI) in predicting outcomes for adolescents and young adults with hematologic malignancies undergoing allogeneic stem cell transplant. Pediatr Blood Cancer. 2011;57:499–505. 10.1002/pbc.23057 21384538

[19] Elfeky R, Builes N, Pearce R, Kania S, Nademi Z, Lucchini G, et al. Pediatric adapted risk index to predict 2-year transplant-related mortality post-HSCT in children. Blood Adv. 2024;8:5838–52. 10.1182/bloodadvances.2024013484 PMC11609366 39093984

[20] Bader P, Salzmann-Manrique E, Balduzzi A, Dalle JH, Woolfrey AE, Bar M, et al. More precisely defining risk peri-HCT in pediatric ALL: pre- vs post-MRD measures, serial positivity, and risk modeling. Blood Adv. 2019;3:3393–405. 10.1182/bloodadvances.2019000449 PMC6855112 31714961

[21] Merli P, Ifversen M, Truong TH, Marquart HV, Buechner J, Wolfl M, et al. Minimal residual disease prior to and after haematopoietic stem cell transplantation in children and adolescents with acute lymphoblastic leukaemia: what level of negativity is relevant? Front Pediatr. 2021;9:777108. 10.3389/fped.2021.777108 PMC8602790 34805054

[22] Segerink WH, de Haas V, Kaspers GJL. Measurable residual disease in pediatric acute myeloid leukemia: a systematic review. Expert Rev Anticancer Ther. 2021;21:451–9. 10.1080/14737140.2021.1860763 33706635

[23] Qayed M, Ahn KW, Kitko CL, Johnson MH, Shah NN, Dvorak C, et al. A validated pediatric disease risk index for allogeneic hematopoietic cell transplantation. Blood. 2021;137:983–93. 10.1182/blood.2020009342 PMC7918183 33206937

