# Supplementary material for: Is the adult EBMT risk score valid and relevant for hematopoietic stem cell transplantation in children acute leukemia?
Source: Bone Marrow Transplant. 2026 Jul 20;61(10):1361–7. doi: 10.1038/s41409-026-02952-8 (PMC13652724; doi:10.1038/s41409-026-02952-8)
Supplement: Supplementary file 1 [file 41409_2026_2952_MOESM1_ESM.docx]

Supplementary Figure 1: Outcomes for transplanted ALL and AML patients according to the EBMT score for the two periods of time 2010-2014 and 2015-2021

Supplementary Figure 1A and 1B represent leukemia-free survival (LFS) for ALL according to the EBMT score for the period 2010-2014 and 2015-2021, respectively. Supplementary Figure 1C and 1D represent LFS for AML according to the EBMT score for the period 2010-2014 and 2015-2021, respectively.
