# Supplementary material for: Is the adult EBMT risk score valid and relevant for hematopoietic stem cell transplantation in children acute leukemia?
Source: Bone Marrow Transplant. 2026 Jul 20;61(10):1361–7. doi: 10.1038/s41409-026-02952-8 (PMC13652724; doi:10.1038/s41409-026-02952-8)
Supplement: Supplementary file 2 [file 41409_2026_2952_MOESM2_ESM.docx]

**Supplementary Table 1: Main features for whole cohort**

| **Features** | **Modalities** | **Whole cohort n=9447 (%)** | **ALL n=5774 (%)** | **AML n=3673 (%)** |
| --- | --- | --- | --- | --- |
| Age at HSCT | median [IQR]  (range) | 9.3 [5.1-13.8]  (0.2-18) | 9.2 [5.7-13.4]  (0.3-18) | 9.6 [3.8-14.3]  (0.2-18) |
| Age at HSCT | [0,4]  [4,10]  [10,18] | 1765 (18.7)  3363 (35.6)  4319 (45.7) | 814 (14.1)  2399 (41.5)  2561 (44.4) | 951 (25.9)  964 (26.2)  1758 (47.9) |
| Year of HSCT | median [IQR]  (range) | 2015 [2012-2018]  (2010-2021) | 2015 [2012-2018]  (2010-2021) | 2015 [2012-2018]  (2010-2021) |
| Months from diagnosis to HSCT | median [IQR]  (range) | 9.1 [5.6-29.7]  (0-193.3) | 17.4 [7.1-39.1]  (0-184.2) | 5.9 [4.4-11.8]  (0.1-193.3) |
| Disease status at HSCT | CR1  CR2  CR>=3  Primary induct fail  Rel1  Rel2  Rel>=3 | 4403 (46.6)  3718 (39.4)  540 (5.7)  274 (2.9)  307 (3.2)  158 (1.7)  47 (0.5) | 2143 (37.1)  2754 (47.7)  486 (8.4)  83 (1.4)  167 (2.9)  101 (1.7)  40 (0.7) | 2260 (61.5)  964 (26.2)  54 (1.5)  191 (5.2)  140 (3.8)  57 (1.6)  7 (0.2) |
| Minimal Residual Disease | Positive  Negative  missing | 743 (16.7)  3712 (83.3)  4206 among 8661 CR | 494 (17)  2410 (83)  2479 among 5383 CR | 249 (16.1)  1302 (83.9)  1727 among 3278 CR |
| Cytogenetic  Acute Myeloid Leukemia classification | Favorable  Intermediate  Adverse  missing |  |  | 436 (16.4)  1258 (47.3)  965 (36.3)  1014 |
| Acute Lymphoblastic Leukemia  lineage | B phi-  B phi not reported  B phi+  T  Other  missing |  | 1139 (20.2)  2553 (45.3)  462 (8.2)  1274 (22.6)  206 (3.7)  140 |  |
| Disease Risk Index | Low  Int  High  Very high | 396 (4.2)  4160 (44)  4400 (46.6)  491 (5.2) | 0 (0)  2143 (37.1)  3240 (56.1)  391 (6.8) | 396 (10.8)  2017 (54.9)  1160 (31.6)  100 (2.7) |
| Hematopoietic Cell Transplantation-Comorbidity index | 0  1 and 2  >=3  missing | 4984 (88.4)  450 (8)  204 (3.6)  3809 | 3092 (89.4)  273 (7.9)  94 (2.7)  2315 | 1892 (86.8)  177 (8.1)  110 (5)  1494 |
| Patient sex | Female  Male | 3702 (39.2)  5745 (60.8) | 2090 (36.2)  3684 (63.8) | 1612 (43.9)  2061 (56.1) |
| Donor sex | Female  Male  missing | 3970 (42.5)  5369 (57.5)  108 | 2411 (42.2)  3304 (57.8)  59 | 1559 (43)  2065 (57)  49 |
| Female to Male | No  Yes | 7089 (75)  2358 (25) | 4268 (73.9)  1506 (26.1) | 2821 (76.8)  852 (23.2) |
| Karnofsky/Lansky score | < 90  >= 90  missing | 1764 (20.4)  6901 (79.6)  782 | 1063 (20.2)  4197 (79.8)  514 | 701 (20.6)  2704 (79.4)  268 |
| Donor CMV | Negative  Positive  missing | 4022 (44.9)  4943 (55.1)  482 | 2491 (45.3)  3004 (54.7)  279 | 1531 (44.1)  1939 (55.9)  203 |
| Patient CMV  Donor to patient CMV | Negative  Positive  missing  Neg to Neg  Neg to Pos  Pos to Neg  Pos to Pos  missing | 3077 (33.6)  6085 (66.4)  285  2102 (23.9)  1830 (20.8)  849 (9.7)  4014 (45.6)  652 | 1902 (33.9)  3702 (66.1)  170  1305 (24.2)  1134 (21)  532 (9.9)  2419 (44.9)  384 | 1175 (33)  2383 (67)  115  797 (23.4)  696 (20.4)  317 (9.3)  1595 (46.8)  268 |
| Source of cells | Bone-marrow  Peripheral Blood  Cord Blood | 4939 (52.3)  3538 (37.5)  970 (10.3) | 3123 (54.1)  2116 (36.6)  535 (9.3) | 1816 (49.4)  1422 (38.7)  435 (11.8) |
| Donor type | MSD  Haplo  UD 10/10  UD 9/10  UD <=8/10  UD (missing HLA)  UCB 6/6  UCB 5/6  UCB <=4/6  UCB (missing HLA) | 2924 (31)  1954 (20.7)  2433 (25.8)  887 (9.4)  72 (0.8)  540 (5.7)  190 (2)  227 (2.4)  40 (0.4)  180 (1.9) | 1792 (31)  1172 (20.3)  1526 (26.4)  543 (9.4)  49 (0.8)  348 (6)  102 (1.8)  130 (2.3)  20 (0.3)  92 (1.6) | 1132 (30.8)  782 (21.3)  907 (24.7)  344 (9.4)  23 (0.6)  192 (5.2)  88 (2.4)  97 (2.6)  20 (0.5)  88 (2.4) |
| EBMT risk score | 0  1  2  3  4  5 | 1129 (12)  2780 (29.4)  1595 (16.9)  2620 (27.7)  1153 (12.2)  170 (1.8) | 522 (9)  1402 (24.3)  973 (16.9)  1862 (32.2)  873 (15.1)  142 (2.5) | 607 (16.5)  1378 (37.5)  622 (16.9)  758 (20.6)  280 (7.6)  28 (0.8) |
| Total Body Irradiation | No  Yes  missing | 5563 (59.2)  3834 (40.8)  50 | 2227 (38.7)  3522 (61.3)  25 | 3336 (91.4)  312 (8.6)  25 |
| Conditioning regimen | BuCy based  BuFlu based  BuFluThio based  BuMel based  Bu+Other  TreoFluThio based  TreoFlu based  Treo+Other  TBI+Cy based  TBI+Eto based  TBI+Flu based  TBI+Mel based  TBI+Other  FluMel based  Other  missing | 1774 (18.9)  590 (6.3)  904 (9.6)  1000 (10.7)  37 (0.4)  666 (7.1)  193 (2.1)  78 (0.8)  1344 (14.3)  1646 (17.6)  675 (7.2)  110 (1.2)  59 (0.6)  195 (2.1)  99 (1.1)  77 | 552 (9.6)  234 (4.1)  639 (11.1)  124 (2.2)  15 (0.3)  388 (6.8)  64 (1.1)  28 (0.5)  1206 (21)  1620 (28.3)  584 (10.2)  68 (1.2)  44 (0.8)  101 (1.8)  65 (1.1)  42 | 1222 (33.6)  356 (9.8)  265 (7.3)  876 (24.1)  22 (0.6)  278 (7.6)  129 (3.5)  50 (1.4)  138 (3.8)  26 (0.7)  91 (2.5)  42 (1.2)  15 (0.4)  94 (2.6)  34 (0.9)  35 |
| Ex-vivo T Cell Depletion | No  Yes  missing | 8072 (87.4)  1160 (12.6)  215 | 4962 (87.9)  685 (12.1)  127 | 3110 (86.8)  475 (13.2)  88 |
| In-vivo T Cell Depletion | No  Yes  missing | 4309 (47.3)  4805 (52.7)  333 | 2576 (46.4)  2980 (53.6)  218 | 1733 (48.7)  1825 (51.3)  115 |
| Post-Transplant Cyclophosphamide | No  Yes  missing | 7952 (90.5)  834 (9.5)  661 | 4839 (90.6)  503 (9.4)  432 | 3113 (90.4)  331 (9.6)  229 |
| GVHD prevention | CSA  CSA+MMF  CSA+MTX  MMF+TACRO  MTX+TACRO  ATG only  Other  missing | 2151 (23.7)  959 (10.6)  4144 (45.7)  300 (3.3)  247 (2.7)  465 (5.1)  808 (8.9)  373 | 1270 (22.9)  524 (9.5)  2622 (47.4)  189 (3.4)  131 (2.4)  301 (5.4)  498 (9)  239 | 881 (24.9)  435 (12.3)  1522 (43)  111 (3.1)  116 (3.3)  164 (4.6)  310 (8.8)  134 |
