# Supplementary material for: Is the adult EBMT risk score valid and relevant for hematopoietic stem cell transplantation in children acute leukemia?
Source: Bone Marrow Transplant. 2026 Jul 20;61(10):1361–7. doi: 10.1038/s41409-026-02952-8 (PMC13652724; doi:10.1038/s41409-026-02952-8)
Supplement: Supplementary file 3 [file 41409_2026_2952_MOESM3_ESM.docx]

**Supplementary Table 2: outcomes**

| **Outcomes** | **Whole cohort**  **(n=9447)** | **AML**  **(n=3673)** | **ALL**  **(n=5774)** |
| --- | --- | --- | --- |
| Median follow-up  (years) | 3 (3 - 3.1) | 3.2 (3.1 - 3.4) | 2.9 (2.8 - 3) |
| Overall Survival  (3 years) | 65.5 (64.4 - 66.6) | 65.5 (63.7 - 67.2) | 65.5 (64.1 - 66.9) |
| Leukemia-Free Survival  (3 years) | 57.3 (56.2 - 58.4) | 58.8 (57.1 - 60.6) | 56.3 (54.8 - 57.7) |
| Relapse Incidence  (3 years) | 30 (29 - 31.1) | 29.7 (28.1 - 31.4) | 30.2 (28.9 - 31.6) |
| Non-Relapse Mortality  (3 years) | 12.7 (12 - 13.4) | 11.4 (10.3 - 12.6) | 13.5 (12.6 - 14.4) |
| Grade III/IV aGVHD  (100 days) | 11.3 (10.7 - 12) | 11.3 (10.2 - 12.3) | 11.4 (10.5 - 12.2) |
| cGVHD  (3 years) | 17.3 (16.4 - 18.2) | 18.2 (16.8 - 19.7) | 16.7 (15.6 - 17.8) |
| GVHD-free Relapse-free survival (3 years) | 45.5 (44.4 - 46.6) | 46.4 (44.6 - 48.2) | 44.9 (43.5 - 46.4) |
