# Supplementary material for: Is the adult EBMT risk score valid and relevant for hematopoietic stem cell transplantation in children acute leukemia?
Source: Bone Marrow Transplant. 2026 Jul 20;61(10):1361–7. doi: 10.1038/s41409-026-02952-8 (PMC13652724; doi:10.1038/s41409-026-02952-8)
Supplement: Supplementary file 4 [file 41409_2026_2952_MOESM4_ESM.docx]

**Supplementary Table 3 : outcomes according to EBMT risk score parameters in ALL and AML**

| **Acute lymphoblastic leukemia** | | | | | | | | | | | | | | | | | | |  | | |  |
| --- | --- | --- | --- | --- | --- | --- | --- | --- | --- | --- | --- | --- | --- | --- | --- | --- | --- | --- | --- | --- | --- | --- |
| **Variables** | **Modalities** | **OS** | | **LFS** | | **RI** | | **NRM** | | **Acute GVH II-IV** | | **Acute III-IV GVHD** | | **chronic GVHD** | | **extensive chronic GVHD** |  | **GRFS** | |  |  |  |
|  |  | **HR**  **(95% CI)** | **p** | **HR (95% CI)** | **p** | **HR (95% CI)** | **p** | **HR (95% CI)** | **p** | **HR (95% CI)** | **p** | **HR (95% CI)** | **p** | **HR (95% CI)** | **p** | **HR (95% CI)** | **p** | **HR (95% CI)** | | **p** |  |  |
| Disease status / time from diagnosis to HSCT | CR1 | 1 |  | 1 |  | 1 |  | 1 |  | 1 |  | 1 |  | 1 |  | 1 |  | 1 | |  |  |  |
|  | CR2 / ≤ 12 months | 1.87  (1.49-2.33) | **<0.001** | 1.88  (1.55-2.29) | **<0.001** | 1.94  (1.53-2.47) | **<0.001** | 1.74  (1.25-2.44) | **<0.001** | 0.8  (0.62-1.03) | 0.08 | 1.32  (0.91-1.92) | 0.14 | 1.35  (0.92-1.98) | 0.12 | 1.37  (0.79-2.37) | 0.26 | 1.82  (1.52-2.18) | | **<0.001** |  |  |
|  | CR2 / > 12 months | 1.38  (1.23-1.55) | **<0.001** | 1.36  (1.23-1.51) | **<0.001** | 1.44  (1.27-1.63) | **<0.001** | 1.23  (1.03-1.46) | **0.02** | 1.05  (0.94-1.16) | 0.39 | 1.13  (0.94-1.35) | 0.19 | 1.28  (1.08-1.50) | **0.003** | 1.3  (1.02-1.64) | 0.03 | 1.34  (1.22-1.46) | | **<0.001** |  |  |
|  | CR3 and active disease / ≤ 12 months | 2.37  (1.84-3.05) | **<0.001** | 2.22  (1.77-2.77) | **<0.001** | 2.74  (2.12-3.54) | **<0.001** | 1.33  (0.84-2.11) | 0.23 | 0.72  (0.52-1.01) | 0.06 | 0.73  (0.41-1.32) | 0.3 | 1.03  (0.62-1.69) | 0.92 | 0.94  (0.44-2.01) | 0.87 | 1.63  (1.32-2.01) | | **<0.001** |  |  |
|  | CR3 and active disease / > 12 months | 2.43  (2.09-2.82) | **<0.001** | 2.25  (1.97-2.57) | **<0.001** | 2.57  (2.19-3.01) | **<0.001** | 1.72  (1.36-2.19) | **<0.001** | 0.86  (0.72-1.02) | 0.09 | 1.00  (0.75-1.32) | 0.99 | 1.10  (0.84-1.44) | 0.48 | 1.16  (0.79-1.72) | 0.44 | 1.81  (1.60-2.05) | | **<0.001** |  |  |
| Donor type | MSD | 1 |  | 1 |  | 1 |  | 1 |  | 1 |  | 1 |  | 1 |  | 1 |  | 1 | |  |  |  |
|  | Non MSD | 1.44  (1.28-1.62) | **<0.001** | 1.22  (1.10-1.34) | **<0.001** | 0.94  (0.84-1.05) | 0.26 | 2.29  (1.88-2.79) | **<0.001** | 1.14  (1.02-1.28) | **0.02** | 1.30  (1.07-1.56) | **0.007** | 0.85  (0.72-0.99) | **0.04** | 0.7  (0.56-0.88) | 0.002 | 1.12  (1.02-1.22) | | **0.01** |  |  |
| Female to Male | No | 1 |  | 1 |  | 1 |  | 1 |  | 1 |  | 1 |  | 1 |  | 1 |  | 1 | |  |  |  |
|  | Yes | 0.98  (0.88-1.09) | 0.67 | 0.93  (0.85-1.03) | 0.16 | 0.9  (0.8-1.02) | 0.09 | 1  (0.84-1.18) | 0.99 | 1.03  (0.93-1.15) | 0.56 | 1.22  (1.02-1.46) | **0.03** | 1.40  (1.20-1.64) | **<0.001** | 1.64  (1.31-2.04) | **<0.001** | 1.04  (0.95-1.13) | | 0.37 |  |  |
| Year of HCT (by 3y increment) | | 0.86  (0.82-0.90) | **<0.001** | 0.95  (0.91-0.99) | **0.007** | 1.03  (0.99-1.09) | 0.17 | 0.79  (0.74-0.85) | **<0.001** | 0.94  (0.90-0.98) | **0.005** | 0.92  (0.85-0.98) | **0.02** | 0.92  (0.86-0.99) | **0.02** | 0.92  (0.86-0.99) | **0.02** | 0.91  (0.87-0.94) | | **<0.001** |  |  |
| *Centre effect taken into account as frailty | |  |  |  |  |  |  |  |  |  |  |  |  |  |  |  |  |  | |  |  |  |
|  |  |  |  |  |  |  |  |  |  |  |  |  |  |  |  |  |  |  | |  |  |  |
|  |  |  |  |  |  |  |  |  |  |  |  |  |  |  |  |  |  |  | |  |  |  |
| **Acute myeloblastic leukemia** | | | | | | | | | | | | | | | | | | |  | | |  |
| **Variables** | **Modalities** | **OS** | | **LFS** | | **RI** | | **NRM** | | **Acute GVH II-IV** | | **Acute III-IV GVHD** | | **chronic GVHD** | | **extensive chronic GVHD** |  | **GRFS** | |  |  |  |
|  |  | **HR (95% CI)** | **p** | **HR (95% CI)** | **p** | **HR (95% CI)** | **p** | **HR (95% CI)** | **p** | **HR (95% CI)** | **p** | **HR (95% CI)** | **p** | **HR (95% CI)** | **p** | **HR (95% CI)** | **p** | **HR (95% CI)** | | **p** |  |  |
| Disease status / time from diagnosis to HSCT | CR1 | 1 |  | 1 |  | 1 |  | 1 |  | 1 |  | 1 |  | 1 |  | 1 |  | 1 | |  |  |  |
|  | CR2 / ≤ 12 months | 1.78  (1.40-2.26) | **<0.001** | 1.75  (1.40-2.18) | **<0.001** | 2.17  (1.70-2.78) | **<0.001** | 0.92  (0.56-1.52) | 0.75 | 0.99  (0.75-1.31) | 0.95 | 0.94  (0.59-1.48) | 0.78 | 1.26  (0.83-1.92) | 0.27 | 0.75  (0.36-1.55) | 0.44 | 1.54  (1.25-1.88) | | **<0.001** |  |  |
|  | CR2 / > 12 months | 1.16  (0.99-1.35) | 0.07 | 1.10  (0.95-1.27) | 0.19 | 1.10  (0.92-1.31) | 0.28 | 1.08  (0.84-1.39) | 0.55 | 0.91  (0.77-1.07) | 0.24 | 0.98  (0.76-1.27) | 0.9 | 1.30  (1.05-1.61) | **0.01** | 0.97  (0.71-1.34) | 0.88 | 1.06  (0.93-1.20) | | 0.41 |  |  |
|  | CR3 and active disease / ≤ 12 months | 3.79  (3.17-4.52) | **<0.001** | 3.71  (3.15-4.37) | **<0.001** | 4.44  (3.68-5.36) | **<0.001** | 2.12  (1.50-2.99) | **<0.001** | 1.40  (1.13-1.75) | **0.002** | 1.60  (1.16-2.23) | **0.005** | 1.48  (1.02-2.15) | **0.04** | 1.55  (0.92-2.62) | 0.1 | 2.85  (2.44-3.33) | | **<0.001** |  |  |
|  | CR3 and active disease / > 12 months | 3.40  (2.65-4.37) | **<0.001** | 3.32  (2.63-4.18) | **<0.001** | 3.30  (2.49-4.38) | **<0.001** | 3.20  (2.16-4.74) | **<0.001** | 1.05  (0.74-1.48) | 0.79 | 1.33  (0.81-2.17) | 0.26 | 1.21  (0.70-2.11) | 0.5 | 1.53  (0.73-3.20) | 0.26 | 2.44  (1.96-3.04) | | **<0.001** |  |  |
| Donor type | MSD | 1 |  | 1 |  | 1 |  | 1 |  | 1 |  | 1 |  | 1 |  | 1 |  | 1 | |  |  |  |
|  | Non MSD | 1.12  (0.98-1.29) | 0.09 | 1.07  (0.95-1.22) | 0.26 | 0.9  (0.78-1.04) | 0.14 | 1.73  (1.35-2.23) | **<0.001** | 1.33  (1.14-1.55) | **<0.001** | 1.40  (1.1-1.79) | **0.006** | 0.94  (0.77-1.15) | 0.54 | 0.91  (0.68-1.21) | 0.5 | 1.08  (0.97-1.21) | | 0.17 |  |  |
| Female to Male | No | 1 |  | 1 |  | 1 |  | 1 |  | 1 |  | 1 |  | 1 |  | 1 |  | 1 | |  |  |  |
|  | Yes | 1.03  (0.9-1.18) | 0.67 | 1.03  (0.90-1.17) | 0.69 | 1.01  (0.87-1.18) | 0.85 | 1.05  (0.83-1.33) | 0.7 | 1.01  (0.87-1.17) | 0.92 | 0.93  (0.73-1.19) | 0.57 | 1.35  (1.11-1.64) | **0.003** | 1.58  (1.20-2.08) | **<0.001** | 1.13  (1.01-1.26) | | **0.04** |  |  |
| Year of HCT (by 3y increment) | | 0.90  (0.85-0.96) | **<0.001** | 0.94  (0.89-0.99) | **0.02** | 1.03  (0.99-1.09) | 0.17 | 0.81  (0.73-0.89) | **<0.001** | 0.89  (0.83-0.94) | **<0.001** | 0.92 (0.85-0.98) | **0.02** | 0.87  (0.79-0.95) | **<0.001** | 0.97  (0.85-1.10) | 0.59 | 0.89  (0.85-0.93) | | **<0.001** |  |  |
| *Centre effect taken into account as frailty | |  |  |  |  |  |  |  |  |  |  |  |  |  |  |  |  |  | |  |  |  |
